# Population-based screening in children for early diagnosis and treatment of familial hypercholesterolemia: design of the VRONI study

**DOI:** 10.1515/medgen-2022-2115

**Published:** 2022-05-07

**Authors:** Veronika Sanin, Raphael Schmieder, Sara Ates, Lea Dewi Schlieben, Jens Wiehler, Ruoyu Sun, Manuela Decker, Michaela Sander, Stefan Holdenrieder, Florian Kohlmayer, Anna Friedmann, Volker Mall, Therese Feiler, Arne Dreßler, Tim M. Strom, Holger Prokisch, Thomas Meitinger, Moritz von Scheidt, Wolfgang Koenig, Georg Leipold, Heribert Schunkert

**Affiliations:** Department of Cardiology, Deutsches Herzzentrum München, Technische Universität München, Lazarettstr. 36, D-80636 Munich, Germany; School of Medicine, Institute of Human Genetics, Technische Universität München, Munich, Germany; Institute of Neurogenomics, Department Computational Health, Helmholtz Zentrum München, Munich, Germany; Bio^M^ Biotech Cluster Development GmbH, Martinsried, Germany; Institute of Laboratory Medicine, Deutsches Herzzentrum München, Technische Universität München, Munich, Germany; Bitcare GmbH, Munich, Germany; Department of Pediatrics, Child and Adolescent Psychosomatics, Technische Universität München, Munich, Germany; Department of Systematic Theology and Ethics, Ludwig-Maximilians-Universität München, Munich, Germany; Deutsches Zentrum für Herz- und Kreislauferkrankungen (DZHK), Partner Site Munich Heart Alliance, Munich, Germany; Professional Association of Pediatricians (BVKJ) of Bavaria, Munich, Germany

**Keywords:** DigiMed Bayern, VRONI, familial hypercholesterolemia, hyperlipidemia, atherosclerosis, screening

## Abstract

Familial hypercholesterolemia (FH) is the most frequent monogenic disorder (prevalence 1:250) in the general population. Early diagnosis during childhood enables pre-emptive treatment, thus reducing the risk of severe atherosclerotic manifestations later in life. Nonetheless, FH screening programs are scarce.

VRONI offers all children aged 5–14 years in Bavaria a FH screening in the context of regular pediatric visits. LDL-cholesterol (LDL-C) is measured centrally, followed by genetic analysis for FH if exceeding the age-specific 95th percentile (130 mg/dl, 3.34 mmol/l). Children with FH pathogenic variants are treated by specialized pediatricians and offered a FH-focused training course by a qualified training center. Reverse cascade screening is recommended for all first-degree relatives.

VRONI aims to prove the feasibility of a population-based FH screening in children and to lay the foundation for a nationwide screening program.

## Background

Familial hypercholesterolemia (FH) represents a common genetic cause of premature coronary heart disease (CHD) and heterozygous FH is the most frequent monogenic disorder in the general population (prevalence 1:250). [1], [2] Nevertheless, FH remains severely underdiagnosed and undertreated worldwide.

The causal genetic variants for FH are mainly located in the LDL receptor (*LDLR*) gene, resulting in a markedly decreased clearance rate of low-density lipoprotein–cholesterol (LDL-C). [3] Other notable genetic variant locations are in the apolipoprotein B (*APOB*) gene, reducing the binding affinity of LDL-C to its receptor (LDLR), and in the proprotein convertase subtilisin/kexin type 9 (*PCSK9*) gene, resulting – in case of a gain-of-function variant – in increased degradation of LDLR in hepatocytes.

FH is clinically characterized by a significant elevation of the LDL-C plasma level and deposition of cholesterol in several tissues. Life threatening may be the premature formation of atherosclerotic plaques, resulting in coronary artery disease (CAD) and cerebrovascular disease in the many affected individuals. [3], [4] Left untreated in the latent stage, individuals with heterozygous FH show a 10-fold increase of cardiovascular risk in their early and middle adulthood. [5], [6], [7] Thus, along with dietary and lifestyle measures, the European guidelines recommend starting statin therapy for FH in childhood. [8], [9]

During childhood serum levels predominantly reflect the genetic component of LDL-C regulation since at this age dietary and hormonal influences are small, thus and offering ideal conditions for FH screening. [5] Moreover, reverse cascade screening offers first-degree relatives of affected children a cost-effective approach for prevention later in life. [10], [11], [12]

VRONI is a multidisciplinary screening program for FH in children aged 5 to 14 years, involving diverse branches within the healthcare sector and logistically and legally supported by a scientific infrastructure.

## Materials and methods

The VRONI study is part of DigiMed Bayern, a pilot project in predictive, preventive, personalized and participatory (P4) medicine in Germany, funded by the Bavarian State Ministry of Health and Care. Multiple fundamental factors were considered during the establishment of VRONI, including ethical, political and legal requirements, data security and privacy, logistics and infrastructure, general acceptance and practicability at the doctor’s office, as well as sustainability and cost efficiency. [13], [14], [15], [16], [17] For instance, special consideration had to be given to data safety, due to the uniquely sensitive nature of genetic datasets of asymptomatic children. VRONI was designed as a proof-of-concept study, offering children in Bavaria a population-based screening program for FH. The **Graphical abstract** provides a conceptual overview of VRONI and the dedicated website www.myVRONI.de offers further information.

### Recruitment

**Figure 1:**
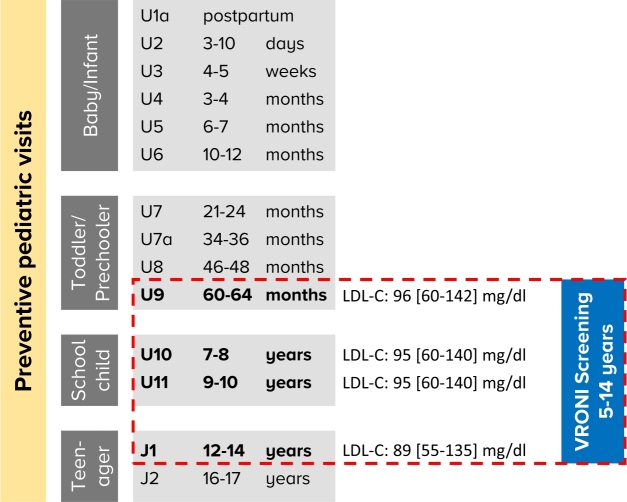
The FH screening by the VRONI study is integrated in an established children examination protocol. The figure shows the recommended preventive pediatric visits in Germany. The VRONI screening period (U9–J1) and the age-specific LDL-C levels measured in the first 5200 participants (median, 5th and 95th percentiles) are outlined in red.

In Germany from birth onwards a structured pediatric screening program is provided for all minors, consisting of partly mandatory preventive medical examinations. These examinations are conducted by pediatricians in their outpatient offices and aim to identify potential diseases early on. However, LDL-C measurements are not part of this routine. In view of practicability and acceptance, VRONI was primarily added to the preventive examinations U9, U10, U11 and J1. Additionally, voluntary enrollment in the context of any patient visit at the pediatricians practice was possible for children aged 5 to 14 years (Figure 1).

In cooperation with the Bavarian Professional Association of Pediatricians (BVKJ) all pediatric doctors in Bavaria were invited to participate in the enrollment of VRONI. Essential documents and laboratory materials were provided by the VRONI main office at the Deutsches Herzzentrum München (DHM). Enrollment consisted of VRONI-specific information material for the child and the parents (or legal guardians) as well as an oral consultation by the attending pediatrician, explaining the study and answering arising questions. After obtaining written informed consent, a blood sample of the child as well as relevant baseline data and cardiovascular family history are collected and mailed to the VRONI main office for further evaluation.

### Screening for individuals at risk and genetic analysis

Guidelines recommend genetic analysis in case of clinical suspicion, aiming to detect a causative variant for FH. [8] In addition to validating the diagnosis of FH, genetic testing can enhance patient management and offers the chance to identify at-risk first-degree relatives via reverse cascade screening.

All VRONI blood samples (200 µl capillary or 1.2 ml venous blood in EDTA) are sent the VRONI main office by mail. LDL-C measurements are then performed by the Institute of Laboratory Medicine at the DHM via a quantitative homogeneous enzyme colorimetric method and are subject to established quality control procedures. To rule out preanalytical errors, for example due to hemolysis, we photometrically measure the indices for hemolysis, icterus and lipemia as a first step. In case of exceeding the thresholds listed in the product information (free hemoglobin <700 mg/dl, conjugated bilirubin <16 g/dl, lipid index <2000) LDL-C measurements are not performed. According to the manufacturer samples below these thresholds LDL-C measurements are not liable to significant deviations.

In case of exceeding the LDL-C threshold of 130 mg/dl, the sample is sent to the Institute of Neurogenomics (ING) at the Helmholtz Zentrum München (HMGU) for genetic analysis in cooperation with the Institute of Human Genetics (IHG) at the Klinikum rechts der Isar der Technischen Universität München (TUM). Based on DNA extracted from the cellular fraction of the initial capillary blood sample, sequencing is conducted on a NovaSeq 6000 (Illumina, CA, USA) at the HMGU and genetic testing utilizes a targeted NGS panel (TWIST Bioscience, CA, USA). This customized FH panel comprises the exonic regions of 23 genes involved in lipid metabolism. Mainly, the whole genomic regions of the *LDLR*, *APOB* and *PCSK9* genes are sequenced, including promoter and intronic regions (but excluding repetitive intronic regions), facilitating detection of potentially disease-relevant non-coding variants. The sequencing data are analyzed and interpretated using the Exome Variant Annotation Database (EVAdb) at the TUM and the genomic database *ClinVar* [18]. For frequency examination of variants, GnomAD is applied. [19]

FH in children is primarily defined as an elevated LDL-C level (>130 mg/dl or >3.34 mmol/l) in conjunction with pathogenic variants in the *LDLR, APOB, PCSK9* and *LDLRAP1* genes. Identification of genetic variants occurs either by classification as “likely pathogenic” or “pathogenic” in *ClinVar* combined with an allele frequency below 0.1 %, or by a loss-of-function mutation (i. e., stop mutation, frameshift mutation, canonical splice shift mutation or large deletion) according to the American College of Medical Genetics and Genomics 2015 guideline. [20]

Of note, causative mutations can only be detected in 60–80 % of clinically definite or probable FH cases. [9] Possible other causes are underlying diseases (e. g., hypothyroidism), epigenetic mechanisms, variants in novel disease genes or a polygenic background, i. e., an accumulation of small effects by a number of single nucleotide polymorphisms (SNPs) along the genome. Therefore, a commonly used screening array (GSA, Illumina, CA, USA) is used to additionally assess the polygenic risk score in all patients with elevated LDL-C levels.

In select cases without pathogenic FH variants but strong clinical suspicion, we will perform whole exome sequencing and/or total mRNA sequencing, aiming to identify FH-associated variants in non-coding regions or genes not yet associated with FH. [21], [22]

The pediatricians receive a scientific genetic report on the findings of known pathogenic variants as well as recommendations and information material for the affected family. In the framework of informing the family about the results (about two to four months after enrollment), a second blood sample (2.7 ml venous blood) is obtained, allowing for central repeat measurement of LDL-C, as generally recommended [2], [23], and repeat genetic testing where appropriate. This procedure is part of the established systematic Failure Mode and Effects Analysis (FMEA) and ensures data quality and minimizes the risk of sample swapping. At the same time a second examination is performed to acquire data on phenotypical FH criteria and/or secondary causes of hypercholesterolemia via a more in-depth family history, a medical history of the child and some data on the parents.

**Figure 2:**
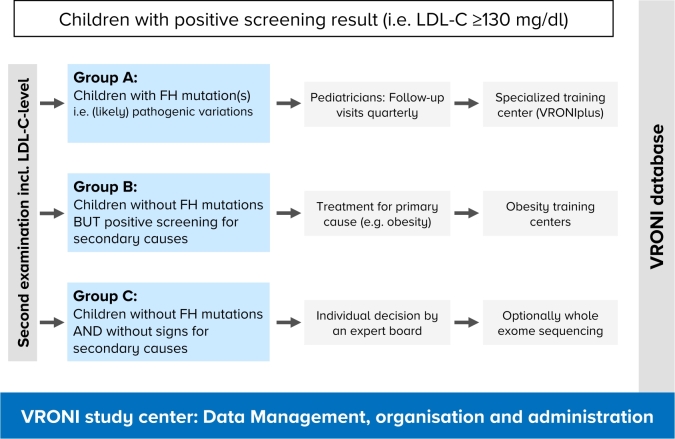
Follow-up in VRONI participants with elevated LDL-C levels. VRONI participants above the LDL-C threshold are subdivided into three distinct groups. Patients in Group A (known pathogenic mutations) receive quarterly follow-up visits by specialized pediatricians or pediatric cardiologists and are offered a FH-focused training course at a specialized training center. Patients in Group B (no known pathogenic mutation, but evidence for secondary causes of hypercholesterolemia) are recommended to be treated accordingly (e. g., referral to specialized obesity training centers in case of obesity). Patients in Group C (no known pathogenic mutation and negative screening for secondary causes) will be reviewed individually by a board of specialists.

**Figure 3:**
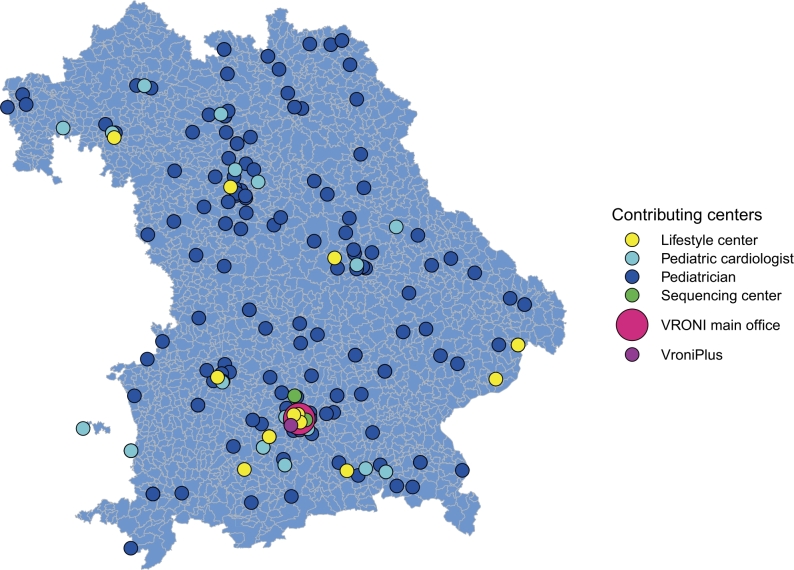
Overview of the VRONI infrastructure in Bavaria. Distribution of contributing centers of the VRONI study in Bavaria. Pink, VRONI main office; green, sequencing center; dark blue, participating pediatricians; light blue, pediatric cardiologists; yellow, preventive lifestyle centers; purple, the VRONIplus training center.

### Follow-up

The additional data of the second examination and blood sample enable the subclassification of children above the LDL-C threshold into three distinct groups: (A) cases with a known pathogenic genetic variant; (B) cases without any known pathogenic genetic variant, but a secondary cause for hypercholesterolemia; and (C) cases without any known pathogenic genetic variant and without a secondary cause for hypercholesterolemia (Figure 2).

(A) All confirmed FH cases are recommended to receive treatment according to the guidelines of the European Society of Cardiology (ESC) and the European Atherosclerosis Society (EAS). [8], [9] Regular follow-ups with routine physical and laboratory examinations are scheduled for Group A, by either the attending pediatrician or a pediatric cardiologist. At baseline an ultrasound examination of the carotid arteries (including intima media thickness measurements) and echocardiography are recommended. VRONI gathers all results of the follow-up visits in the form of dedicated questionnaires, including laboratory and diagnostic findings.
(B) After identifying the (likely) secondary cause for hypercholesterolemia, the VRONI main office informs the attending pediatrician, who in turn initiates the appropriate treatment of the primary cause (e. g., obesity, hypothyroidism, nephrotic syndrome, anorexia nervosa, etc.). In case of obesity, a referral to a specialized obesity training center (in Bavaria there are 12 centers) is recommended (Figure 3).
(C) Group C calls for an individual review of each case, which will be carried out by a board of experts in the fields of cardiology, lipidology, genetics and pediatric cardiology. Afterwards the attending pediatrician receives recommendations regarding possible follow-up visits, pharmacotherapy, or further diagnostics (entire exome sequencing or functional analysis in selected cases). Lifestyle modifications are indicated in all children of Group C.

VRONI focuses on children with FH (Group A) and offers a range of additional assistance to affected families and the attending pediatricians. The first major challenge, for children especially, is to comprehend and accept the diagnosis of a “lifelong” disease such as FH. Moreover, the diagnosis represents a huge psychological challenge not only for the child, but for the parents as well. VRONIplus offers every affected family professional support, primarily in the form of a FH-focused training session at a specialized training center. The goal is to educate the children in a suitable manner and provide the rest of the family with the tools to implement and most notably maintain recommended lifestyle changes and pharmacotherapy. VRONIplus is a one-day psychoeducational program developed by professional social pediatrics. An accompanying website deepens and re-addresses important items posttraining to facilitate the transfer of learned knowledge into everyday life. Additionally, a weekly psychosocial online consultation (via video chat) with an experienced psychologist offers the family advice regarding family stresses or acute issues in connection with FH. In addition, VRONI created a dedicated booklet for children, explaining FH and the recommended treatment by the way of a cartoon character affected by FH. Overall, the care of FH patients requires a multidisciplinary team and collaboration between pediatricians, pediatric cardiologists, nutritionists and psychological experts.

Aiming to detect other affected family members, VRONI offers testing for related minors as part of the VRONI study. In cooperation with CaRe High, a patient registry and a cascade screening program for FH in Germany, adult family members of affected children are offered further testing in the form of a reverse cascade screening. [24]

Of note, the genetic analysis in VRONI, performed within the framework of a research project, only qualifies as a scientific report and does not equate legally to a valid medical diagnosis in Germany. Consequently, for a final diagnosis a confirmation (of the highly suspected FH diagnosis) by conventional focused genotyping in accordance with the German health insurance regulations is needed. This has the benefit of ruling out any mix-up or analysis errors, however a genetic diagnosis can also be a stigma, which may for instance prohibit or hinder some insurance policies.

### IT infrastructure and data protection

Considering the sensitive nature of genetic and medical data, the cornerstone of VRONI’s IT infrastructure is data security and privacy. The database was developed in a modular design, enabling excellent protection of identifiers and easy collection, processing and integration of large amounts of disparate data at the same time. For safety reasons all analytical procedural steps are performed exclusively on pseudonymized data. VRONI includes the use of various IT technologies, e. g., statistical and analytical tools such as knowledge management systems, to maximize process security and efficiency as well as output. Data quality is monitored via data flow monitoring at various stages to ensure appropriate quality standards. The VRONI data protection and security concept was developed in accordance with and approved by the responsible data protection authorities and local ethics committees.

## Comments

The VRONI study is the first population-based screening program using routine genetic testing for the early detection of FH in children in Bavaria, Southern Germany. Screening started in January 2021 and the goal is to enroll 50,000 children and younger adolescents within a period of three years.

### Diagnosis and screening

Generally, diagnosis of FH is handled non-uniformly, with no single internationally accepted set of criteria for a clinical diagnosis. The most commonly used criteria are derived from lipid clinic registries (Simon-Broome diagnostic criteria [25], Dutch Lipid Clinic Network criteria [26], Make early Diagnosis to Prevent early Deaths [27], or Japanese Atherosclerosis Society [28]). However, diagnosing FH in younger individuals is somewhat difficult, because commonly used lipid scores are not applicable in children since clinical features, such as increased Achilles tendon thickness, are quite uncommon or rather not yet manifested at a young age.

The Fr1dolin study in Lower Saxony, Germany [13] represents a clinical approach for early detection of Hypercholesterolemia in children, focusing on the clinical phenotype (LDL-Hypercholesterolemia) via LDL-C measurements without routine genetic testing. However, it remains unclear which LDL-C cut-off should be used for FH detection in children. Moreover, no valid clinical score for diagnosing FH in children exists. Thus, the guidelines clearly recommend genetic testing in case of clinical suspicion to confirm FH via detection of causative genetic variants. In conclusion FH screening in children requires a comprehensive strategy incorporating clinical features as well as genetic testing. In this respect, VRONI’s aim is to contribute to the development of a children-specific clinical FH score and to offer new data and insights regarding the ongoing debate on who, when and how to screen for FH and when and how to treat children with FH.

In 2011 the National Lipid Association recommended that universal screening should be carried out at age 9–11 years when lipid changes associated with puberty are less evident and statin therapy can be started. [29] On the other hand, Wald et al. demonstrated the feasibility of a child–parent screening in children aged 1–2 years via capillary cholesterol measurement during routine immunization combined with reverse cascade screening of parents, detecting four children and four parents with FH every 1000 children screened. [30] Irrespective of the exact time frame and method, experts agree that childhood is the optimal period for discrimination between FH and non-FH using LDL-C screening. [5] In this respect, universal screening for FH in the context of pediatric routine visits seems to be the ideal strategy to identify children with FH.

### Screening needs a multidisciplinary approach

Different studies have shown that there are several gaps in the knowledge, perception and practice in FH among general practitioners from different countries. [31], [32] Hence, pediatricians in particular need to be familiar with the diagnosis, treatment and management of FH and should be actively involved in the screening process. Thus, VRONI established screening at local pediatric offices in conjunction with additional genetic analysis of samples with elevated LDL-C levels, supporting the development of a sequential strategy of biochemical screening followed by targeted sequencing.

Previous results of an International Pediatric FH Register (funded by the International Atherosclerosis Society, IAS), including data on country-specific as well as common characteristics of the management of FH in childhood across Europe, revealed that the majority of children are lacking the full benefit of early FH identification and advantages of guideline-directed therapy. [33] Notably, only a small fraction of FH cases is appropriately treated. However, not only FH diagnosis, but also management and treatment of FH need broad recognition to realize the potential positive impact on public health. In addition to medication, patient support groups and specialized training centers are fundamental in improving the care of children with FH and their families, which is offered in the form of the VRONIplus study. In view of the co-dominant inheritance of FH, identifying and treating the index patient is only the first step, followed by cascade screening of first-degree relatives. VRONI collaborates with CaRe High to offer genetic testing and counseling to all families affected by FH, which is strongly recommended as a cost-effective and fundamental part of FH diagnosis in recent guidelines. [34], [35] Indeed, countries where dedicated cascade screening programs are implemented have identified a notably higher number of patients with FH. For instance, the Netherlands and Norway have diagnosed 71 % and 43 % of FH cases, respectively. [36] In this context primary care seems to be a key target area to increase identification of new index cases and initiate cascade screening in a second step.

Overall key aspects of improving diagnosis and treatment of FH seem to be spreading the awareness and knowledge about the disease itself, implementing screening to diagnose FH early on and having a multidisciplinary treatment approach for catching potential psychological issues and ensuring a sufficient long-term therapy starting in childhood.

### Treatment: “the younger, the better”

Patients with FH are considered to be at high or very high risk for premature CHD. The severity of atherosclerosis and premature CHD in FH is proportional to the cumulative burden of LDL-C levels. [37] Therefore, effective and aggressive cholesterol treatment early on, favorably beginning with a high-intensity statin, could reduce the LDL-C burden and the risk for cardiovascular disease. VRONI offers treatment recommendations and collects baseline and long-term follow-up data of children with FH, including clinical data, laboratory parameters and genetic data, for further treatment.

### Ethical issues

In the context of genetically testing children within the VRONI study, important ethical issues arise. Moreover, genetic test results also provide information about close relatives, resulting in implications for them as well, which further complicate the individual’s fundamental right “(not) to know.” We address these issues with an additional sociological research project and investigate how participants and pediatricians actually understand and handle VRONI’s chances and challenges at the same time.

**Table 1:** Predefined main research outcomes of VRONI. Overview of the predefined main aims of the VRONI study.

**Clinical features and health economics management**
– Data analyses and reporting of the registry data on estimated prevalence, geographical distribution, single genetic variants associated with disease and polygenic scores. – Identification of clinical criteria helpful for the diagnosis of FH. – Evaluation of a collaboration between families and physicians. – Value from a public health perspective, cost effectiveness and acceptance of a generalized screening. – Cost effectiveness and treatment strategies including an interventional approach in the context of reverse cascade screening.
**Treatment (lifestyle and LDL-C lowering medication)**
– Changes in lipid parameters. – Changes in carotid intima media thickness. – Changes in measures of growth and maturation. – Identification of parameters to monitor treatment. – Role of additional lifestyle risk factors (obesity, low leisure time physical activity, high blood pressure and smoking) in prevention. – Analyses of long-term side effects on treatment with statins or ezetimibe. – Long-term cost/benefit analysis of treatment.
**Patient outcomes**
– Screening and treatment effects on long-term risk of cardiovascular events. – Quality of life assessment. – Treatment compliance and adherence.

### Implication and perspective

In this respect the VRONI study will usefully contribute to complementing the comprehensive model of care for FH, offering insights gained from the development of VRONI’s complex multidisciplinary network as well as the epidemiologic and clinical data collected. Findings can also inform healthcare regulators regarding the rationale for genetic testing for FH, recommendations for clinical practice and guidelines for management of FH. Validation and implementation of a practicable systematic FH screening approach, such as VRONI, is an important challenge that we aim to address in this study, hopefully paving the way for a standardized, highly qualitive and health-economically cost-efficient system of care for FH. Additionally, we will address a number of research outcomes comprising the interplay between genetic and lifestyle risk factors, clinical features, health economics management, treatment and outcomes (Table 1). This multidisciplinary screening approach allow us to discover potential benefits regarding advancements in human genetics and personalized medicine. Combining recent advancements in the field of digital technology based on Big Data (electronic health records, FH registries, clinical databases), it also seems feasible to establish efficient tools for FH detection and implementation in public health systems with relatively low efforts. VRONI will also be linked to the EAS and the Familial Hypercholesterolemia Studies Collaboration (FHSC), which aims to establish a worldwide, large-scale and standardized registry of patients with FH, containing data on detection strategies and the clinical implications thereof. [38]

## Summary and conclusion

Population-based screening for FH seems a simple, practical and effective way to identify and prevent a common cause of premature cardiovascular death. In collaboration with primary care pediatricians, VRONI will evaluate the feasibility of a population-based FH screening in children aged 5–14 years in the context of routine check-ups. VRONI aims to incorporate clinical and molecular diagnostics into establishing a systematic effort for diagnosing individuals with FH, followed by a multidisciplinary treatment approach, spanning patient education and counseling as well as regular follow-up assessments. By routine screening of children and reverse cascade screening of first-degree relatives, FH can be diagnosed before the onset of cardiovascular events, enabling to treat preventively. FH should be treated early and intensively, because children and adolescents with FH who started treatment early show a similar prognosis as those without FH. [7] Furthermore, VRONI will record a large population-based dataset on FH individuals, allowing for better assessment of the FH prevalence in Southern Germany and providing essential data for improving overall care and support for FH patients as well as for developing national or international FH screening programs. Also, the data evaluation will certainly help to improve new diagnostic tools in risk stratification for personalized medicine. Aside from that, VRONI will raise and hopefully elucidate important ethical questions in the context of genetic diseases like FH. Overall, VRONI’s main goal is to implement FH screening into the standard screening for inherited metabolic diseases in children (e. g., phenylketonuria and homocystinuria), which is already part of the routine care for children in Germany. VRONI also provides important data regarding the trend to a personalized approach, such as using genotype, lifestyle and environmental factors for tailoring treatment.

## Supplementary Material

Bavarian Pediatricians Consortium

DigiMed Bayern Consortium
