# Supplementary material for: Population-based screening in children for early diagnosis and treatment of familial hypercholesterolemia: design of the VRONI study
Source: Med Genet. 2022 May 7;34(1):41–51. doi: 10.1515/medgen-2022-2115 (PMC11006262; doi:10.1515/medgen-2022-2115)
Supplement: Supplementary file 1 — Bavarian Pediatricians Consortium [file medgen-2022-2115suppa.docx]

*Bavarian Pediatricians Consortium:

Renate Abt, Wendelstein, Germany; Judith Aderbauer, Weiden i.d. OPf, Germany; Bettina Aichholzer, Bad Endorf, Germany; Ina Albrich, Dorfen, Germany; David Antos, Traunstein, Germany; Tina Arenz, Pfaffenhofen, Germany; Markus Ascher, Dingolfing, Germany; Sabine Auerochs, Nürnberg, Germany; Bernd Aulinger, Burglengenfeld, Germany; Monika Aumer-Hauke, Naila, Germany; Andrea Bachmeyr, München, Germany; Albert Baier, Donauwörth, Germany; Georg Baier, Schwabach, Germany; Yvonna Bauer, Augsburg, Germany; Bastian Baumgartner, Regensburg, Germany; Larissa Baumgärtner, Heilsbronn, Germany; Wolfgang Beck, Donauwörth, Germany; Mariella Becker, Kempten, Germany; Elisabeth Beer, Marktredwitz, Germany; Sabine Beer, Amberg, Germany; Sonja Behrendt, München, Germany; Lutz Bellingrath, Bamberg, Germany; Rüdiger Graf von Bentzel, Augsburg, Germany; Marcus Benz, Dachau, Germany; Rita Berger, Straubing, Germany; Alexander Bernhart, München, Germany; Ralf Beyrich, Langenzenn, Germany; Christoph Bidlingmaier, München, Germany; Katrin Biebach, München, Germany; Matthias Bierler, Amberg, Germany; Helena Biermann-Franke, Erlangen, Germany; Stefanie Blum, Erding, Germany; Sabine Böhm-Vogt, Ottobrunn, Germany; Milena Bornkamm, München, Germany; Karola Börzsönyi, Freising, Germany; Stephanie Bosch, Deggendorf, Germany; Nadja Bösel, Neumarkt, Germany; Ralf Brangenberg, Traunstein, Germany; Daniel Breitfeld, München, Germany; Johannes Breuninger, Mindelheim, Germany; Wolfgang Brosi, Würzburg, Germany; Edeltraud Burg, Straubing, Germany; Bianca Cantori, Eggenfelden, Germany; Mona Castrop, Regensburg, Germany; Alina Claudia Cocos, Markt-Schwaben, Germany; Monika Corbacioglu, Regensburg, Germany; Daniela Diebel, Hof, Germany; Lutz Dietrich, Hof, Germany; Dietrich Distel, Neustadt a.d. Aisch, Germany; Barbara Domes, Karlsfeld, Germany; Jürgen Dörrer, Freyung, Germany; Carmen Dümmler, Nürnberg, Germany; Stefan Eber, München, Germany; Heinrich Eberhardt, Landshut, Germany; Iris Eckmüller, Pfarrkirchen, Germany; Ulrike Ege-Mirzai, Bamberg, Germany; Thomas Eichinger, Bernhardswald, Germany; Annette Eiden, Gauting, Germany; Tobias Eisenhut, München, Germany; Hans-Dieter Eisner, Kronach, Germany; Uta Enzensberger, Mering, Germany; Patricia Erlinger, Postbauer-Heng, Germany; Dominik A. Ewald, Regensburg, Germany; Kirsten Exl, München, Germany; Florian Fackler, Erlangen, Germany; Martin Falke, Straubing, Germany; Daniela Felsl, Wolnzach, Germany; Doris Feulner-Kamleitner, Lichtenfels, Germany; Magdalena Fick, Landshut, Germany; Maria Fischer, Lappersdorf, Germany; Cordula Fischer-Trüstedt, München, Germany; Roland Frank, Coburg, Germany; Harald Frantzmann, Memmingen, Germany; Monika Freiwald-Tries, Schonungen, Germany; Claudia Frey, Augsburg, Germany; Georg Fröhlich, Mindelheim, Germany; Thorsten Fröhlich, Forchheim, Germany; Monika Gaigl, Erding, Germany; Susanne Gandenberger-Bachem, München, Germany; Nadine Geda, Bayreuth, Germany; Elke Gerlitz, Herzogenaurach, Germany; Jürgen Geuder, Freilassing, Germany; Taraneh Ghassemi-Keller, Goldbach, Germany; Thomas Gilb, Augsburg, Germany; Johannes Gilles, Gunzenhausen, Germany; Silvia Glotzbach-Sack, Würzburg, Germany; Birgit Goldschmitt-Wuttge, München, Germany; Christine Götz, Geretsried, Germany; Martin Götz, Elisabethszell, Germany; Martin Griebel, Poing, Germany; Anne Grieger, Roth, Germany; Britta Groschup, Haibach, Germany; Dietrich Grunert, Kempten, Germany; Florian Gundel, Starnberg, Germany; J. Peter Gutdeutsch, Regensburg, Germany; Sonja Habash, Cham, Germany; Thomas Habash, Cham, Germany; Henrik Halboni, München, Germany; Emma Halwas, Geisenfeld, Germany; Michael Hardt, München, Germany; Matthias Hartig, Arnstorf, Germany; Christian Hartnik, Bad Staffelstein, Germany; Anna Hasche-Trebin, Bad Staffelstein, Germany; Matthias Hasenpusch, Erding, Germany; Marita Haslinger, München, Germany; Gabi Haus, München, Germany; Johannes Hausmann, Würzburg, Germany; Anja Hauth, Schonungen, Germany; Rudolf Havla, Hof, Germany; Ralph Heidingsfelder, Ansbach, Germany; Simone Heier, Stadtbergen, Germany; Alfred Heihoff, Regensburg, Germany; Beatrice Heineking, Geretsried, Germany; Barbara Helmreich, Schweinfurt, Germany; Markus Helmreich, Schweinfurt, Germany; Amalia Herineanu, Nürnberg, Germany; Holger Hertzberg, Schwabach, Germany; Daniela Heuschmann, Regensburg, Germany; Klaus Hoffmann, Niederwern, Germany; Rolf Höfler, Kempten, Germany; Andrea Högl, Schwandorf, Germany; Sabina Hohn, Nürnberg, Germany; Alexandra Holzapfel, Landsberg am Lech, Germany; Reinhard Hopfner, Prien a. Chiemsee, Germany; Jan-Helge Höpner, Eckental, Germany; Daniela Hoppen, München, Germany; Jörg Horcher, Straubing, Germany; Michael Horn, Schönau a.K., Germany; Stephan von Hornstein, Olching, Germany; Birte Marei Huber, Erding, Germany; Benedikta Huber-Lederer, Fürstenzell, Germany; Katrin Hucke, Nürnberg, Germany; Wolfgang Hüttner, Coburg, Germany; Armin Irnstetter, Geretsried, Germany; Stephan Jarosch, Würzburg, Germany; Elke Sabine Jenne, München, Germany; Eckhard Jentsch, Weißenburg, Germany; Ania Jocham, München, Germany; Benedikt Jochem, Lappersdorf, Germany; Birgit Jork-Käferlein, Prien a. Chiemsee, Germany; Guido Judex, Regensburg, Germany; Ronny Jung, Roth, Germany; Andreas Kalmutzki, Schwabach, Germany; Michael Andreas Kandler, Nürnberg, Germany; Torsten Kaussen, Pocking, Germany; Jens Keppler, Amberg, Germany; Cornelia Kiani, Bayreuth, Germany; Christa-Maria Kitz, Veitshöchheim, Germany; Annegret Klein, Oberaudorf, Germany; Julia Klein, München, Germany; Kristina Klemp, Bad Staffelstein, Germany; Daisy Klimm-Pozo, Schwabmünchen, Germany; Margreth Knebel, München, Germany; Hans-Werner Knüppel, Rothenburg, Germany; Margit Kosoko, Vilsbiburg, Germany; Margarethe Kozuschek, Würzburg, Germany; Maike Kral, Haibach, Germany; Renate Kramer, München, Germany; Barbara Krappatsch, Viechtach, Germany; Dörte Kreutz, Langenzenn, Germany; Annika Kurzhals, Weißenhorn, Germany; Dominik Kutter, Augsburg, Germany; Franz Lachner, Ruhpolding, Germany; Christof Land, Gauting, Germany; Carolin Lang, Bad Tölz, Germany; Martin Lang, Augsburg, Germany; Manuel Langer, Würzburg, Germany; Otto Laub, Rosenheim, Germany; Michaela Lebok, Bad Staffelstein, Germany; Georg Leipold, Regensburg, Germany; Egbert Leonhardt, Weiden i.d. OPf, Germany; Katrin Leuchtenberger, Kelheim, Germany; Thay Mi Le-Weimer, Nürnberg, Germany; Caroline Lewerenz, Traunstein, Germany; Hans G. Lichtenstern, Pocking, Germany; Sabrina Lindner, Regensburg, Germany; Ruth Lindner-Gajek, München, Germany; Ulf Lüdicke, Rödental, Germany; Renata Lysy, Möhrendorf, Germany; Brigitta Macé, Eggenfelden, Germany; Silvia Maier, Gilching, Germany; Brigitte Maier-Brandt, Stein, Germany; Georg Mair, Bad Endorf, Germany; Soyoun Maisch, München, Germany; Stephan Martini, München, Germany; Monika Maurus, Memmingen, Germany; Barbara Meiler, Grafrath, Germany; Udo Meißner, Bamberg, Germany; Volker Melichar, Neunkirchen, Germany; Steffi Menzel, München, Germany; Susanne Merget, Germering, Germany; Roland Metzner, Würzburg, Germany; Angelika Meyer, Nürnberg, Germany; Ingeborg Meyer, Fürth, Germany; Oliver Michael, Murnau, Germany; Miriam Mrach, München, Germany; Udo Mulitze, Mainburg, Germany; Arnd Müller, München, Germany; Patrick Muzzolini, Kulmbach, Germany; Stefanie Naegele, München, Germany; Dieter Nagel, Grafenau, Germany; Gerhard Nagel, Forchheim, Germany; Andrea Nestler, Markt Indersdorf, Germany; Klaus Neumann, Höhenkirchen, Germany; Nuria Nöbel, Stein, Germany; Jochen Noss, München, Germany; Jutta Oberndorfer, Schonungen, Germany; Michael Pätzold, Marktoberdorf, Germany; Christian Pauli, Olching, Germany; Matthias Sigmund Peisler, Forchheim, Germany; Angela Pfeffer, Regen, Germany; Beatrix Pfirstinger, Abensberg, Germany; Julia Pistohl, Deggendorf, Germany; Roman Polanetz, München, Germany; Heike Polster, Dingolfing, Germany; Andreas Pontz, Passau, Germany; Simone Porz, Neuburg a.d. Donau, Germany; Thomas Potthast, Kempten, Germany; Manfred Praun, Gilching, Germany; Constanze Preis, Bischberg, Germany; Wolfgang Preis, Bischberg, Germany; Stephanie Putzker, München, Germany; Axel Quattländer, Volkach, Germany; Gertraud Raber-Webhofer, München, Germany; Angela Rausch, Traunstein, Germany; Markus Redenbacher, Nürnberg, Germany; Anja Regenfus, Nürnberg, Germany; Christoph Reiber, Friedberg, Germany; Fabian Reif, Nürnberg, Germany; Denise Reimnitz, Goldbach, Germany; Evelyn Reineke, Karlshuld, Germany; Anne-Kathrin Reinhardt, Bernhardswald, Germany; Tobias Reinhardt, Feuchtwangen, Germany; Mustafa Rihawi, Kronach, Germany; Jan Ripper, Grafrath, Germany; Anke Robert, Neuburg a.d. Donau, Germany; Julia Roeb, Starnberg, Germany; Wolfram Rohland, Erding, Germany; Melanie Römer, Kempten, Germany; Ulrich Römer, Traunstein, Germany; Manfred Rösch, Nürnberg, Germany; Mark Rosenthal, Unterschleißheim, Germany; Florina Rössler, Herzogenaurach, Germany; Christian Rudolf, Bad Neustadt, Germany; Ramon Rümler, Dachau, Germany; Uwe L. Sack, Würzburg, Germany; Reinhard Sailer, Vilsbiburg, Germany; Norbert Salih, München, Germany; Franziska Schaaff, Eckental, Germany; Frank Scharnowski-Fischer, Weiden i.d. OPf, Germany; Carolus Schenke, Neustadt a.d. Aisch, Germany; Harald Scheuermeyer, Haibach, Germany; Heinrich Schiegl, Schierling, Germany; Ute Schindler, Kelheim, Germany; Christian Schirl, Neustadt a.d. Aisch, Germany; Hubert Schirmer, Marktredwitz, Germany; Lydia Schlak, Sulzbach-Rosenberg, Germany; Andreas Schloßbauer, Bad Kissingen, Germany; Hubert Schmid, Pfarrkirchen, Germany; Roland Schmid, Bruckmühl, Germany; Petra Schmid-Seibold, Regensburg, Germany; Volker Schmidt, Kempten, Germany; Birte Schmitt, Erlangen, Germany; Beate Schneider, Roth, Germany; Sofie Schneider, Gersthofen, Germany; Martin Schöniger, Weilheim, Germany; Philipp Schoof, München, Germany; Thomas Schuch, Dachau, Germany; Eleonore von der Schulenburg, München, Germany; Carola Schum, Parsberg, Germany; Andrea Schürmann, Hof, Germany; Brigitte Schwager, Eckental, Germany; Annika Schwarz, Gilching, Germany; Stefan Schwarz, Bayreuth, Germany; Christine Schweikl, Eggenfelden, Germany; Rosemarie Schwertner, Germering, Germany; Regina Schwindler, Nabburg, Germany; Julia Andrea Seemann, Deggendorf, Germany; Florian Segerer, Regensburg, Germany; Monika Seidt, München, Germany; Nina Sellerer, München, Germany; Marko Senjor, Wasserburg, Germany; Katrin Seybold, Roth, Germany; Brigitte Simmendinger, Aschaffenburg, Germany; Eva-Kristina Soballa-Stehr, Trostberg, Germany; Anita Sommer, Karlsfeld, Germany; Wiebke Specht, Pfersee, Germany; Claudia Spieß, Neuburg a.d. Donau, Germany; Karsten Stahnke, Günzburg, Germany; Kerstin Stecker, Weißenhorn, Germany; Lidia Steigerwald, Hof, Germany; Kathrin Steins, Wolfrathshausen, Germany; Stefan Stellwag, München, Germany; Olga Stepanowa, Bayreuth, Germany; Heiko Stern, Gauting, Germany; Anke Steuerer, Augsburg, Germany; Dominik Stricker, Lappersdorf, Germany; Michael Strobelt, Bruckmühl, Germany; Raphael Sturm, Affing, Germany; Hans Georg Terbrack, Abensberg, Germany; Wolfgang Theil, Gersthofen, Germany; Günter Theurer, Traunstein, Germany; Zühal Tomas, Gersthofen, Germany; Burkhard Trusen, Bamberg, Germany; Birgit Turba-Eckhardt, Landshut, Germany; Peter Unger, Burghausen, Germany; Johannes Josef Urban, Augsburg, Germany; Michael Veh-Hölzlein, Fürth, Germany; Hartmut Vogel, Roth, Germany; Christian Voigt, Stadtbergen, Germany; Roland Wagner, Nittendorf, Germany; Lorens Wajswasser, Wendelstein, Germany; Karin Waldmann, Memmelsdorf, Germany; Ulrich Warweg, Grassau, Germany; Anna Weber, Würzburg, Germany; Annette Weber-Pöhlmann, Selb, Germany; Johannes Weigel, Augsburg, Germany; Christina Weigmann-Popp, Bamberg, Germany; Kirsten Wenner, Diessen, Germany; Beatrix Wenzel, Lichtenfels, Germany; Robert Werner, Landau, Germany; Christine Wieland, München, Germany; Oliver Wiese, Landsberg am Lech, Germany; Margit Wiessner-Straßer, München, Germany; Konrad Alois Wimmer, Passau, Germany; Jochen Winkler, Schwabmünchen, Germany; Tobias Winter, Teisendorf, Germany; Marco Wölfel, Bayreuth, Germany; Olga Wolfram, Herzogenaurach, Germany; Michaela Wruk, Bad Endorf, Germany; Lothar Wurzer, Oberstdorf, Germany; Aytac Yaman, Lichtenfels, Germany; Caroline Zeches-Kansy, Haag i. Obb, Germany; Alexander Zeiss, München, Germany; Stefan Zeller, Memmingen, Germany; Sabine Zenger, Herzogenaurach, Germany; Mathias Zimmer, Coburg, Germany; Thomas Zimmermann, Hirschaid, Germany; Stefan Zink, Nürnberg, Germany; Britta Zötl, Bad Tölz, Germany; Gregor Zuj, Schwandorf, Germany; Kristin Zwenzner, Bayreuth, Germany;
