# Supplementary material for: Population-based screening in children for early diagnosis and treatment of familial hypercholesterolemia: design of the VRONI study
Source: Med Genet. 2022 May 7;34(1):41–51. doi: 10.1515/medgen-2022-2115 (PMC11006262; doi:10.1515/medgen-2022-2115)
Supplement: Supplementary file 2 — DigiMed Bayern Consortium [file medgen-2022-2115suppb.docx]

***DigiMedBayernConsortium:**

Reiner Anselm, Institut Technik – Theologie – Naturwissenschaften, Ludwig-Maximilians-Universität München, Munich, Germany; Sara Ates, Deutsches Herzzentrum München, Technische Universität München, Munich, Germany; Sabine Bauer, Deutsches Herzzentrum München, Technische Universität München, Munich, Germany; Nicole Beck, Deutsches Herzzentrum München, Technische Universität München, Munich, Germany; Jürgen Beckmann, Fakultät für Sport- und Gesundheitswissenschaften, Technische Universität München, Munich, Germany; Stefan Brandmaier, Deutsches Herzzentrum München, Technische Universität München, Munich, Germany; Salvatore Cassese, Deutsches Herzzentrum München, Technische Universität München, Munich, Germany; Manuela Decker, Deutsches Herzzentrum München, Technische Universität München, Munich, Germany; Martin Dichgans, Institut für Schlaganfall- und Demenzforschung , Technische Universität München, Munich, Germany; Philine Diesselhorst, Juristische Fakultät, Universität Augsburg, Augsburg, Germany; Horst Domdey, BioM Biotech Cluster Development GmbH, Martinsried, Germany; Martina Dreßen, Deutsches Herzzentrum München, Technische Universität München, Munich, Germany; Arne Dressler, Institut Technik – Theologie – Naturwissenschaften, Ludwig-Maximilians-Universität München, Munich, Germany; Florent Dufour, Leibniz-Rechenzentrum, Munich, Germany; Sven Duscha, Deutsches Herzzentrum München, Technische Universität München, Munich, Germany; Gertrud Eckstein, Institut für Humangenetik, Technische Universität München, Munich, Germany; Therese Feiler, Institut Technik – Theologie – Naturwissenschaften, Ludwig-Maximilians-Universität München, Munich, Germany; Anton Frank, Leibniz-Rechenzentrum, Munich, Germany; Ulrich M. Gassner, Juristische Fakultät, Universität Augsburg, Augsburg, Germany; Philipp Geyer, Max-Planck-Institut für Biochemie, Munich, Germany; Christian Gieger, Institut für Epidemiologie, Helmholtz Zentrum München, Munich, Germany; Elisabeth Graf, Institut für Humangenetik, Technische Universität München, Munich, Germany; Ulrich Güldener, Deutsches Herzzentrum München, Technische Universität München, Munich, Germany; Nicolay Hammer, Max-Planck-Institut für Biochemie, Munich, Germany; Johann Hawe, Deutsches Herzzentrum München, Technische Universität München, Munich, Germany; Thomas Hendel, Institut für Epidemiologie, Helmholtz Zentrum München, Munich, Germany; Stephan Jonas, Institut für Informatik, Technische Universität München, Munich, Germany; Adnan Kastrati, Deutsches Herzzentrum München, Technische Universität München, Munich, Germany; Wolfgang Kempf, Klinikums rechts der Isar, Technische Universität München, Munich, Germany; Thorsten Keßler, Deutsches Herzzentrum München, Technische Universität München, Munich, Germany; Wolfgang Koenig, Deutsches Herzzentrum München, Technische Universität München, Munich, Germany; Florian Kohlmayer, Bitcare, Munich, Germany; Markus Krane, Deutsches Herzzentrum München, Technische Universität München, Munich, Germany; Dieter Kranzlmüller, Institut für Informatik, Ludwig-Maximilians-Universität München, Munich, Germany; Harald Lahm, Deutsches Herzzentrum München, Technische Universität München, Munich, Germany; Rüdiger Lange, Deutsches Herzzentrum München, Technische Universität München, Munich, Germany; Andreas Lehmann, Bitcare GmbH, Munich, Germany; Ling Li, Deutsches Herzzentrum München, Technische Universität München, Munich, Germany; Peter Lichtner, Institut für Humangenetik, Technische Universität München, Munich, Germany; Birgit Linkohr, Institut für Epidemiologie, Helmholtz Zentrum München, Munich, Germany; Sandy Lösecke, Institut für Humangenetik, Technische Universität München, Munich, Germany; Lars Maegdefessel, Klinikums rechts der Isar, Technische Universität München, Munich, Germany; Matthias Mann, Max-Planck-Institut für Biochemie, Munich, Germany; Thomas Meitinger, Institut für Humangenetik, Technische Universität München, Munich, Germany; Irina Neb, Deutsches Herzzentrum München, Technische Universität München, Munich, Germany; Shichao Pang, Deutsches Herzzentrum München, Technische Universität München, Munich, Germany; Annette Peters, Institut für Epidemiologie, Helmholtz Zentrum München, Munich, Germany; Roland Pichler, Max-Planck-Institut für Biochemie, Munich, Germany; Paola Pisano, Max-Planck-Institut für Biochemie, Munich, Germany; Holger Prokisch, Institut für Humangenetik, Technische Universität München, Munich, Germany; Lara Marie Reimer, Institut für Informatik, Technische Universität München, Munich, Germany; Veronika Sanin, Deutsches Herzzentrum München, Technische Universität München, Munich, Germany; Niklas Schleicher, Institut Technik – Theologie – Naturwissenschaften, Ludwig-Maximilians-Universität München, Munich, Germany; Lea Dewi Schlieben, Institut für Humangenetik, Technische Universität München, Munich, Germany; Yannick Schlote, Institut Technik – Theologie – Naturwissenschaften, Ludwig-Maximilians-Universität München, Munich, Germany; Sofie Schmid, Klinikums rechts der Isar, Technische Universität München, Munich, Germany; Raphael Schmieder, Deutsches Herzzentrum München, Technische Universität München, Munich, Germany; Heribert Schunkert, Deutsches Herzzentrum München, Technische Universität München, Munich, Germany; Lisa Schweizer, Max-Planck-Institut für Biochemie, Munich, Germany; Megi Sharikadze, Leibniz-Rechenzentrum, Munich, Germany; Fabian Starnecker, Deutsches Herzzentrum München, Technische Universität München, Munich, Germany; Sophia Steigerwald, Max-Planck-Institut für Biochemie, Munich, Germany; Tim-Matthias Strom, Institut für Humangenetik, Technische Universität München, Munich, Germany; Ruoyu Sun, BioM Biotech Cluster Development GmbH, Martinsried, Germany; Fernando Cross Villasana, Institut für Informatik, Technische Universität München, Munich, Germany; Moritz von Scheidt, Deutsches Herzzentrum München, Technische Universität München, Munich, Germany; Annie Westerlund, Deutsches Herzzentrum München, Technische Universität München, Munich, Germany; Jens Wiehler, BioM Biotech Cluster Development GmbH, Martinsried, Germany; Michael Wierer, Max-Planck-Institut für Biochemie, Munich, Germany; Peter Zinterhof, Leibniz-Rechenzentrum, Munich, Germany;
